# Insights into genetic modifiers of breast cancer risk in carriers of *BRCA1* and *BRCA2* pathogenic variants

**DOI:** 10.1186/s13053-025-00313-y

**Published:** 2025-04-28

**Authors:** Roksana Dwornik, Katarzyna Białkowska

**Affiliations:** Department of Genetics and Pathology, International Hereditary Cancer Center, Pomeranian Medical University, Szczecin, Poland

## Abstract

Pathogenic variants in *BRCA1* and *BRCA2* are associated with an increased risk of developing several types of cancer, including breast cancer. However, the risk varies by other environmental and genetic factors present in carriers of mutation. To understand the value of these factors more clearly, a number of common genetic susceptibility variants have been studied through genome-wide association studies as potential genetic risk modifiers for *BRCA1* and *BRCA2* pathogenic variants carriers. Several studies have identified specific polymorphisms that may influence the risk of breast cancer development, either by increasing or reducing susceptibility. These variants are implicated in biological pathways such as DNA damage repair, hormonal regulation or cell proliferation. The identification and understanding of key genetic modifiers may provide valuable insights into development of personalized prevention, targeted therapies and screening strategies for high-risk individuals. This review presents the overview of known genetic risk modifiers for carriers of *BRCA1* and *BRCA2* pathogenic variants, their potential impact on risk, and their functional roles. Furthermore, it highlights the need for further research directions, including understanding the biological role of genetic modifiers in cancer development and the refinement of risk assessment models.

## Introduction

Breast cancer is the most common cancer in women worldwide. Approximately 2,3 million women are diagnosed with breast cancer each year [1]. It ranks as the fifth leading cause of cancer-related deaths globally, accounting for 685,000 fatalities [2]. Pathogenic variants in the *BRCA1* and *BRCA2* genes are associated with increased risk of developing breast cancer. The life-time risk of breast cancer for carriers of *BRCA1* and *BRCA2* pathogenic variants is about 65% and 45%, respectively [3, 4]. This risk can be influenced by a variety of specific factors. There are numerous studies investigating the impact of reproduction and environmental factors on the cancer penetrance among *BRCA1/2* mutation carriers [5–8]. Moreover, common genetic variants have also been reported to modify that penetrance [9–30]. As a result, women with the same mutation may develop cancer – or remain unaffected – depending on the additional genetic variants they carry. The aim of this study is to review and summarize the existing data on genetic modifiers of breast cancer risk in female *BRCA1* and *BRCA2* pathogenic variants carriers. It also emphasizes the value of research focused on genetic modifiers.

## Methodology

The literature search was conducted in November 2024, and articles published from 2003 to 2024 were included in the review. The search terms comprised ‘BRCA1 modifiers breast cancer’, ‘BRCA2 modifiers breast cancer’, ‘BRCA1 genetic modifiers’, ‘BRCA2 genetic modifiers’, ‘BRCA1 modifiers GWAS’, ‘BRCA2 modifiers GWAS’, ‘BRCA1 CIMBA’, ‘BRCA2 CIMBA’. Boolean operator ‘AND’ was used to combine terms. A search was conducted using the PubMed database.

**Fig. 1:**
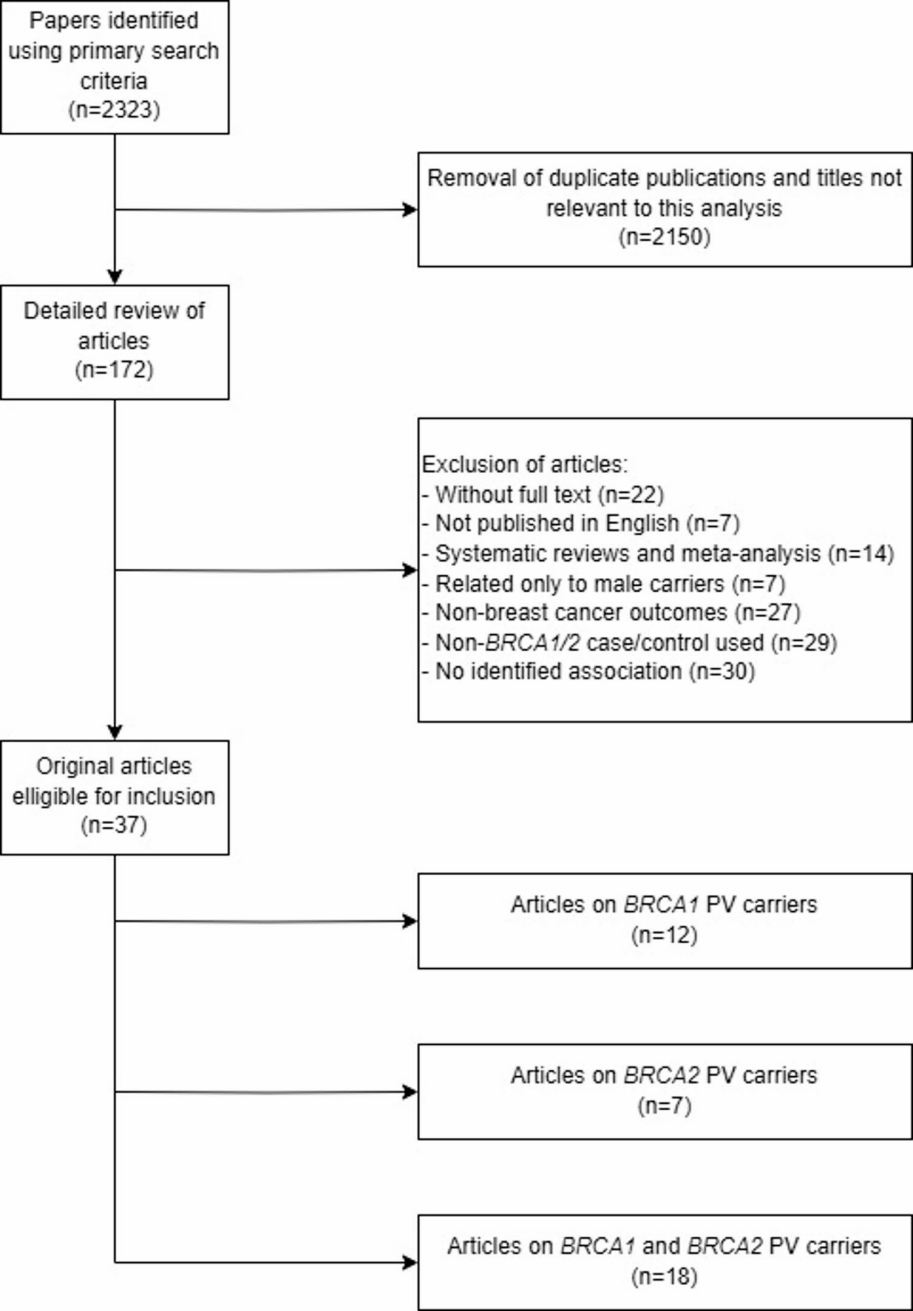
Strategy used to identify literature about genetic modifiers

The detailed results of literature search strategy are shown in Fig. 1. A systematic search of papers was conducted according to established criteria to identify studies on genetic modifiers affecting breast cancer risk in carriers of PV in the *BRCA1* and *BRCA2* genes. The initial literature search found 2323 articles that met the basic search criteria. At the first stage, duplicates and articles unrelated to the topic of the study were eliminated, resulting in the removal of 2150 papers. The remaining 172 articles were subjected to a detailed evaluation, which resulted in the exclusion of publications that did not meet the specified inclusion criteria. Articles not available in full text and papers published in a language other than English were rejected. In addition, review articles and meta-analyses were excluded to focus on original research. Furthermore, papers that analyzed only male PV carriers and articles focusing on outcomes unrelated to breast cancer were excluded. Studies that used non-*BRCA1/2* cases/controls were also removed, as well as papers that did not show any significant association between the analyzed genes and cancer risk.

Finally, the compilation of the 37 selected articles formed the basis of this review, and the results of these studies were presented to highlight the genetic modifiers of breast cancer risk in carriers of PV in the BRCA1 and BRCA2 genes. The analysis of these studies provided a better understanding of how various genetic factors can modify the risk of developing breast cancer in this group of individuals.

### Genome wide-association studies

A number of common polymorphisms in candidate genes have been studied as a potential factors that may modify breast cancer risk in carriers of *BRCA1* and *BRCA2* pathogenic variants. These studies have focused on genes considered functionally significant for the disease or those that interact with *BRCA1* and/or *BRCA2* genes. Knowledge of these risk modifiers could enable the more specific prediction of breast cancer progression in mutation carriers. Furthermore, they may result in the development of new therapies [31].

**Fig. 2:**
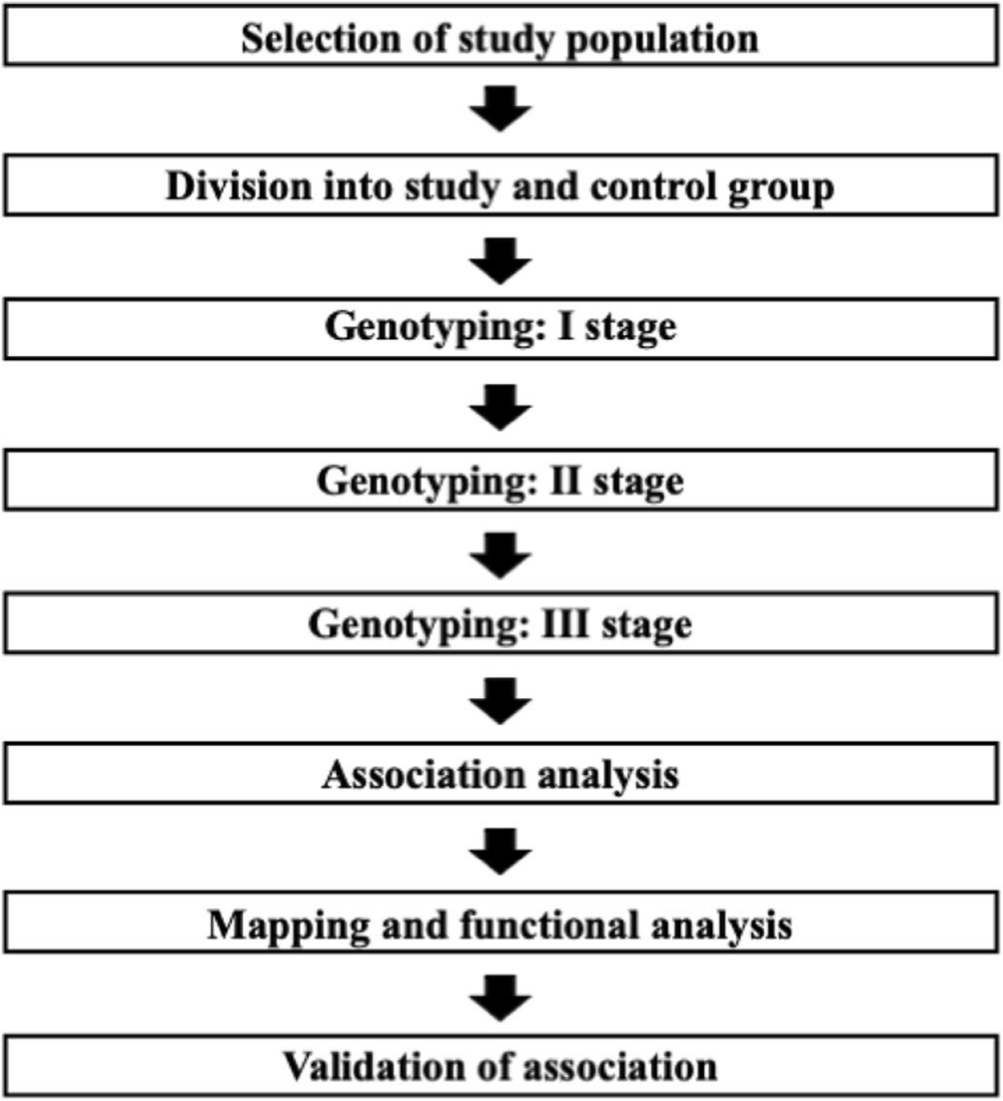
Steps of conducting GWAS

Previous large-scale association studies conducted by the Consortium of Investigators of Modifiers of *BRCA1* and *BRCA2* (CIMBA) have provided evidence of such breast cancer risk modifiers [31]. These studies examined common genetic variants which have been identified through genome-wide association studies (GWAS) as being linked to breast cancer risk in general population [32–34]. A genome-wide association study proceed in several steps (Fig. 2) [35, 36]. Through GWAS, copy-number variants (CNVs) or sequence variations in the human genome can be analysed, although single-nucleotide polymorphisms (SNPs) constitute the most frequently studied genetic variants in such studies [35]. GWAS is most often conducted by using pre-existing resources – disease-specific cohorts or biobanks. The selected cohort is divided into study and control group. The homogeneity of the study group in terms of the analysed feature is. Genotyping of individuals is usually performed using microarrays for common variants or, less frequently, using next-generation sequencing methods – whole exome sequencing (WES) or whole genome sequencing (WGS). Genotyping is carried out in several stages. First, in “discovery study”, a small proportion of the samples from cases and controls are tested. Then, SNPs that show the most significant associations with disease risk are retested in subsequent studies involving larger group. In the third phase, the study group expands significantly and may consist of tens of thousands of participants. After three phases of genotyping, the SNPs showing the strongest association are selected as markers that may influence disease. Typically, in GWAS, association testing is done by using linear or logistic regression models. Markers selected in study are further evaluated by mapping and performing functional studies to assess the association with the disease. The preliminary association should be replicate in an independent cohort. The last stage of study focuses on the validation of detected associations. The standard significance threshold for GWAS is *p*-value of 5.0 × 10^− 8^ [37].

### Genetic variants associated with breast cancer risk for *BRCA1* pathogenic variants

The risk of developing breast cancer in carriers of pathogenic variants in the *BRCA1* gene may be caused not only by the occurrence of mutation, but also by genetic modifiers. There is evidence that specific variants in some genes may influence the penetrance of breast cancer in *BRCA1* mutation carriers. Numerous studies have focused on investigating SNPs which are located within genes being important in cellular processes, such as the regulation of cell growth or DNA repair [38].

One of the SNPs examined by CIMBA was a polymorphism of the apoptosis-related gene *CASP8* (rs1045485). It was found that carriers of mutations in the *BRCA1* gene with the ‘CC’ genotype at this locus have a reduced risk of breast cancer [39]. Another study showed that SNP c.1298 A > C in *MTHFR* gene may also reduce the risk. Genotypes ‘AC’ and ‘CC’ were associated with two-fold decreased breast cancer risk in Polish women carrying *BRCA1* mutations [22]. Other genes whose polymorphisms have been identified as associated with a lower risk of breast cancer in *BRCA1* mutation carriers are *ANKLE1* (rs2363956), *SNRPB* (rs6138178), *VEGF* (rs3025039), *TERT* (rs2180341) and *PTHLH* (rs10771399) (Table 1).

**Table 1:** SNPs found to be associated with reduced breast cancer risk for *BRCA1* pathogenic variants carriers

Gene	Locus	SNP	Sample size	Unaffected/ Affected	HR (95% CI)	*P*-value	Genotyping platform	Function	Ref
*CASP8*	2q33	rs1045485	4844	2241/2603	0.85 (0.76–0.97)	0.028	iPLEX, TaqMan	Cell apoptosis regulation	[39]
*MTHFR*	1p36	rs1801131	457	225/232	0.64 (0.51–0.80)	< 0.0001	RFLP-PCR	Metabolism of folate and regulation of homocysteine level	[22]
*ANKLE1*	19p13.11	rs2363956	7517	3367 ⁄ 4150	0.84 (0.80–0.89)	5.5 × 10^− 9^	Illumina	DNA damage response	[16]
*SNRPB*	20p13	rs6138178	3451	1540/1911	0.78 (0.69–0.90)	3.6 × 10^− 4^	Illumina	Component of spliceosome, involved in mRNA splicing	[29]
*VEGF*	6p21.3	rs3025039	457	225/232	0.63 (0.41–0.98)	0.042	PCR-RFLP	Angiogenesis induction	[23]
*TERT*	6q22.33	rs2180341	3361	1642/1719	0.89 (0.80-1.00)	0.048	TaqMan, iPLEX	Maintaining telomere length	[24]
*PTHLH*	12p11	rs10771399	12 558	6190/6368	0.87 (0.81–0.94)	3.2 × 10^− 4^	iPLEX	Regulation of bone and cartilage development	[12]
*BRCA1-*wild type	17q21.31	rs16942	7048	3481/3567	0.86 (0.77–0.95)	0.003	TaqMan, iPLEX	DNA repair, cell cycle regulation, tumour suppressor	[19]

There is evidence that benign variants in *BRCA1* may also modify cancer risk among *BRCA1* PV carriers. Cox et al. showed that women with the rare allele of SNP rs16942 on the wild-type copy of *BRCA1* exhibited a reduced risk of breast cancer [19]. Another study reported that an intron variant of *BRCA1* (rs5820483) is associated with exon 11 isoform expression, alternative splicing and the risk of developing breast cancer in *BRCA1* PV carriers. Ruiz de Garibay et al. confirmed that effect in mouse cells, suggesting that disruption of *BRCA1* exon 11 splicing modifies the cancer risk linked to pathogenic *BRCA1* variants [40].

Variants increasing breast cancer risk have also been investigating. The ‘T’ allele of the SNP c.1630 C > T in *PHB* (rs6917) has been found to be associated with a two-fold increased breast cancer risk in Polish population [41]. The SNP rs6602595 in *CAMK1D* gene have also been reported as a modifier increasing breast cancer risk in *BRCA1* pathogenic variants carriers [29]. There is also evidence that a non-synonymous polymorphism in *IRS1* modifies breast cancer risk among *BRCA1* PV carriers. Ding et al. reported that the variant *of IRS1* (rs1801278), which interacts with insulin-like growth factor (IGF1R) and insulin receptor (IR), is associated with two-fold increased risk of developing breast cancer in *BRCA1* class 2 mutation carriers [42]. Polymorphisms in the *BABAM1* (rs8170), *TERT* (rs10069690), *TCF7L2* (rs11196174), *MDM4* (rs2290854), *MTHFR* (rs1801133) *and ESR1* (rs2046210, rs9397435) likewise may increase the risk of breast cancer (Table 2).

**Table 2:** SNPs found to be associated with higher breast cancer risk for *BRCA1* pathogenic variants carriers

Gene	Locus	SNP	Sample size	Unaffected/ Affected	HR (95% CI)	*P*-value	Genotyping platform	Function	Ref
*PHB*	17q21	rs6917	516	258/258	2.12 (1.23–3.70)	0.006	RFLP-PCR	Mitochondrial integrity, transcriptional modulation	[41]
*CAMK1D*	10p3	rs6602595	3451	1540/1911	1.25 (1.10–1.41)	4.2 × 10^− 4^	Illumina	Involvement in the calcium signalling pathway; neuronal signalling	[29]
*IRS1*	2q36	rs1801278	577	231/346	1.86 (1.28–2.70)	0.0011	TaqMan, iPLEX	Involvement in insulin signalling, regulating glucose metabolism	[42]
*BABAM1*	19p13.1	rs8170	11 669	3755/3870	1.20 (1.13–1.28)	8.7 × 10^− 9^	iPLEX	DNA repair-dependent chromatin remodelling	[43]
*TERT*	5p15.33	rs10069690	11,705	11,705*	1.23 (1.16–1.29)	1.6 × 10^− 14^	iCOGS	Maintaining telomere length	[44]
*TCF7L2*	10q25.3	rs11196174	14 346	7035/7311	1.13 (1.07–1.18)	7.5 × 10^− 7^	iCOGS, iPLEX	Influencing the transcription, especially in the Wnt signalling	[18]
*MDM4*	1q32	rs2290854	14 350	7037/7313	1.13 (1.08–1.18)	1.4 × 10^− 7^	iCOGS, iPLEX	Inhibition of p53 activity	[18]
*ESR1*	6q25.1	rs2046210	10 817	5302/5515	1.17 (1.11–1.23)	4.5 × 10^− 9^	iPLEX	Mediating the effect of the estrogen on various target tissues	[11]
*ESR1*	6q25.1	rs9397435	12 575	6201/6374	1.28 (1.18–1.40)	1.3 × 10^− 8^	iPLEX	Mediating the effect of the estrogen on various target tissues	[11]
*MTHFR*	1p36	rs1801133	457	225/232	2.69 (1.80–4.02)	< 0.0001	RFLP-PCR	Metabolism of folate and regulation of homocysteine level	[22]

*No detailed information provided; this information concerns the total number of *BRCA1* mutation carriers participating in the study without division into unaffected/affected

Breast cancer in individuals with *BRCA1* PV is primarily ER-negative [45]. As a result, SNPs associated with ER-positive breast cancer in the general population, which account for most susceptibility variants identified through GWAS, are unlikely to affect the risk in *BRCA1* PV carriers. Therefore, several studies have examined the association of genetic modifiers with the risk of tumour subtypes defined by ER-status. Notably, associations with ER-negative tumours—but not ER-positive tumours—have been confirmed for rs8170 in *BABAM1*, rs67397200 in *BABAM* [43], rs1045485 in *CASP8* [46] and rs3817198 in *LSP1* [47], among others (Table 3).

**Table 3:** SNPs found to be associated with Estrogen receptor status in breast cancer among *BRCA1* and *BRCA2* pathogenic variants carriers

BRCA1 mutation carriers							ER-positive		ER-negative					
Gene	**Locus**	**SNP**	**Sample size**	**Unaffected**	**ER+**	**ER-**	**HR (95% CI)**	***P***	**HR (95% CI)**	***P***		**Genotyping platform**		**Ref**
*BABAM1*	19p13.1	rs8170	6844	4483	541	1820	1.12 (0.96–1.29)	0.15	**1.23 (1.14–1.33)**	**2.0 × 10** ^**− 7**^		iPLEX		[43]
*BABAM1*	19p13.1	rs67397200	6849	4486	542	1821	1.14 (1.01–1.30)	0.04	**1.22 (1.14–1.30)**	**4.4 × 10** ^**− 9**^		iPLEX		[43]
*CASP8*	2q33	rs1045485	60 128	36 976	17 805	5347	0.96 (0.93-1.00)	0.06	**0.90 (0.84–0.96)**	**0.001**		Illumina, iPLEX		[46]
*LSP1*	11p15.5	rs3817198	8628	3996	438	1435	1.07 (0.93–1.22)	0.33	**1.07 (1.00-1.15)**	**0.047**		iPLEX, TaqMan		[47]
*BRCA2* mutation carriers														
*FGFR2*	10q26.13	rs2981582	4738	2102	841	263	**1.35 (1.23–1.48)**	**1.4 × 10** ^**− 10**^	1.14 (0.97–1.35)	0.12		iPLEX, TaqMan		[47]
*TOX3/* *TNRC9*	16q12.1	rs3803662	4563	2037	815	255	**1.28 (1.16–1.41)**	**1.5 × 10** ^**− 6**^	1.06 (0.88–1.29)	0.53		iPLEX, TaqMan		[47]
*LSP1*	11p15.5	rs3817198	5354	2332	1022	320	**1.17 (1.07–1.28)**	**5.5 × 10** ^**− 4**^	1.03 (0.81–1.22)	0.70		iPLEX, TaqMan		[47]
*SLC4A7/NEK10*	3p24.1	rs4973768	5669	2528	1108	329	**1.13 (1.04–1.22)**	**0.0043**	1.02 (0.88–1.19)	0.78		iPLEX, TaqMan		[47]

Even though SNPs are the primary focus of genetic modifier studies, copy number variants (CNVs) are also considered in such research, but their contribution is relatively unknown. Recent study suggests that deleterious variants in *SULT1A1* may alter the breast cancer risk in carriers of *BRCA1* mutation. The findings show that deletions in *SULT1A*, a gene encoding sulfotransferase 1A1 responsible for catalyse the sulfate conjugation of hormones, drugs and xenobiotics, may reduce the risk in *BRCA1* PV carriers [48]. Moreover, another genome-wide CNVs analysis have reported that deletions in *GTF2H2* are linked to a reduced risk of breast cancer. Since GTF2H2 is involved in nucleotide excision repair (NER), this result suggests that NER disruption may provide protection against the effects of a *BRCA1* pathogenic variants [49].

For *BRCA1* PV carriers, polymorphisms in *ANKLE1* (rs2363956), *BABAM1* (rs8170), *TERT* (rs10069690) and *ESR1* (rs2046210, rs9397435) reached GWAS significance threshold (*p*-value < 5.0 × 10^− 8^).

### Genetic variants associated with breast cancer risk for *BRCA2 *pathogenic variants

Breast cancer risk associated with mutations in the *BRCA2* gene, as with the *BRCA1* gene, can be altered by genetic modifiers. In addition, for the *BRCA2*, more genes variants have been identified that may influence breast cancer penetrance in mutation carriers.

Several studies have shown a modifying effect of the *RAD51* c.135G > C (rs1801320) polymorphism on the risk of breast cancer in carriers of pathogenic variants in *BRCA2* [14, 50]. It has been found that mutation carriers with ‘CC’ genotype at this locus are at three-fold increased risk of developing breast cancer compared with the ‘GG’ genotype [14]. Another study presents that the variants in *TOX3/TNRC9* (rs3803662) and *FGFR2* (rs2981582) may also increase the risk of breast cancer in *BRCA2* mutation carriers. Moreover, it has been considered that for the combined effect of the two loci, the absolute risk of developing disease ranges from 41% for individuals with no risk alleles to 70% for those carrying four risk alleles [15]. It also has been proven that common variant in *ALDH2* (rs10744777) may modify the lifetime risk of breast cancer for *BRCA2* mutation carriers. Recent study has shown that the ‘TT’ genotype of the *ALDH2* (rs10744777) variant combined with the *BRCA2* p.K3326* variant increases the breast cancer risk among carriers by 1,72-fold [30]. There is likewise evidence that carriers with both *BRCA1/2* pathogenic variants and mutations in *PPARGC1A*, a gene involved in energy metabolism regulation, may develop breast cancer at a significantly younger age [51]. An association with higher risk of developing breast cancer in *BRCA2* pathogenic variants carriers has also been demonstrated for the polymorphisms in *LSP1* (rs3817198), *MAP3K1* (rs889312), *LOC134997* (rs9393597) and *FBXL7* (rs12652447). There is additionally evidence of association for SNPs in *SMAD3* (rs3825977, rs7166081), *EMBP1* (rs11249433), *SLC4A7/NEK10* (rs4973768), *FGF10/MRPS30* (rs10941679), *FGF13* (rs619373) and *ESR1* (rs9397435) (Table 4).

**Table 4:** SNPs found to be associated with higher breast cancer risk for *BRCA2* pathogenic variants carriers

Gene	Locus	SNP	Sample size	Unaffected/ Affected	HR (95% CI)	*P*-value	Genotyping platform	Function	Ref
*RAD51*	15q15.1	rs1801320	2748	1174/1574	3.18 (1.39–7.27)	0.0004	TaqMan, RFLP-PCR	DNA repair through homologous recombination	[14]
*TOX3/* *TNRC9*	16q12.1	rs3803662	3255	1426/1829	1.15 (1.03–1.27)	0.009	iPLEX, TaqMan	Chromatin remodelling	[15]
*FGFR2*	10q26.13	rs2981582	3260	1427/1833	1.32 (1.20–1.45)	1.7 × 10^− 8^	iPLEX, TaqMan	Cell growth and differentiation	[15]
*ALDH2*	12q24.12	rs10744777	19 488	11,873/7615	1.72 (1.19–2.48)	0.003	TaqMan	Detoxification of acetaldehyde	[30]
*LSP1*	11p15.5	rs3817198	5434	2404/3030	1.16 (1.07–1.25)	2.8 × 10^− 4^	iPLEX,, TaqMan	Immune cell signalling and adhesion	[13]
*MAP3K1*	5q11.2	rs889312	3524	1557/1967	1.12 (1.02–1.24)	0.020	iPLEX, TaqMan	Regulation of cell migration	[15]
*LOC134997*	6q22	rs9393597	2006	887/1119	1.55 (1.25–1.92)	6.0 × 10^− 5^	Illumina	Non-coding RNA (IncRNA) family, regulation of gene expressions	[29]
*FBXL7*	5p15.1	rs12652447	2006	887/1119	1.37 (1.16–1.62)	1.7 × 10^− 4^	Illumina	Regulation of protein degradation	[29]
*SMAD3*	15q22	rs3825977	2693	1189/1504	1.20 (1.03–1.40)	0.018	Illumina, iPLEX	TGF- β signalling pathway	[28]
*SMAD3*	15q22	rs7166081	2693	1189/1504	1.25 (1.07–1.45)	0.004	Illumina, iPLEX	TGF- β signalling pathway	[28]
*EMBP1*	1p11.2	rs11249433	6250	2827/3423	1.09 (1.02–1.17)	0.015	iPLEX,, TaqMan	IcnRNA class, regulation of gene expression	[11]
*SLC4A7/* *NEK10*	3p22	rs4973768	6153	2783/3370	1.10 (1.03–1.18)	6.4 × 10^− 3^	iPLEX, TaqMan	Ion transport, protein phosphorylation	[10]
*FGF10/* *MRPS30*	5p12	rs10941679	5854	2591/3263	1.09 (1.01–1.19)	0.032	iPLEX TaqMan	Cell growth, regulation of mitochondrial function	[10]
*ESR1*	6q25.1	rs9397435	7117	3313/3804	1.14 (1.01–1.28)	0.031	iPLEX, TaqMan	Mediating the effect of the estrogen on various target tissues	[11]
*FGF13*	Xq27.1	rs619373	8207	3881/4326	1.30 (1.17–1.45)	3.1 × 10^− 6^	iCOGS	Embryonic development, cell growth	[21]

In a recent study it has been found that a truncating variant of *RAD52* (rs4987207) is significantly associated with reduced breast cancer risk in *BRCA2* mutation carriers [9]. Moreover, the *RAD52 S346X* variant has been identified as reducing double-strand break (DSB) repair through the single strand annealing (SSA) pathway. The present findings may impact future cancer treatment and they suggests that inhibitors of RAD52 could be potentially used to reduce the risk of breast cancer in *BRCA2* pathogenic variant carriers [9]. The inverse association between a breast cancer risk in carriers of *BRCA2* mutation and the presence of a given SNP in another gene has been also observed for a polymorphism in *ZNF365* (rs16917302), albeit not at a genome-wide level of significance. The association of this SNP was statistically significant in stage 1 of study but not in stage 2, although in combined analysis of stage 1 and stage 2, the ‘C’ allele was associated with reduced risk of developing breast cancer in *BRCA2* pathogenic variants carriers [20]. Evidence of risk-modifying factors for breast cancer in carriers of *BRCA2* mutation also indicates polymorphisms in other genes, such as *ABL1* (rs3808814), *CYP1B1-AS1* (rs184577), *TFAP2A* (rs9348512), *LOC105376214* (rs865686), *GMEB2* (rs311499) and *ZNF365* (rs10995190) (Table 5).

**Table 5:** SNPs found to be associated with reduced breast cancer risk for *BRCA2* pathogenic variants carriers

Gene	Locus	SNP	Sample size	Unaffected/ Affected	HR (95% CI)		*P*-value	Genotyping platform	Function	Ref
*RAD52*	12p13.33	rs4987207	10 979	5374/5605		0.69 (0.56–0.86)	8.0 × 10^− 4^	Illumina	DNA double-strand break repair	[9]
*ZNF365*	10q21.2	rs16917302	4188	2026/2162		0.75 (0.66–0.86)	3.8 × 10^− 5^	Affymetrix, iPLEX	Regulation of gene expression, involvement in the cell cycle	[20]
*ZNF365*	10q21.2	rs10995190	7119	3315/3804		0.90 (0.82–0.98)	0.015	iPLEX	Regulation of gene expression, involvement in the cell cycle	[12]
*ABL1*	9q34.12	rs3808814	2693	1189/1504		0.71 (0.53–0.97)	0.030	Illumina, iPLEX	Tyrosine kinase activity	[28]
*CYP1B1-AS1*	2p22.2	rs184577	8211	3881/4330		0.85 (0.79–0.91)	3.6 × 10^− 6^	iCOGS	IncRNA class, regulation of gene expression	[21]
*TFAP2A*	6p24	rs9348512	6214	3881/2333		0.85 (0.80–0.90)	3.9 × 10^− 8^	iCOGS	Transcriptional regulation	[21]
*LOC105376214*	9q31.2	rs865686	7111	3312/3799		0.95 (0.89–1.01)	7.3 × 10^− 3^	iPLEX	IncRNA class, regulation of gene expression	[12]
*GMEB2*	20q13.3	rs311499	4138	2001/2137		0.72 (0.61–0.85)	6.6 × 10^− 5^	Affymetrix, iPLEX	Modulation of glucocorticoid receptors activity	[20]

Moreover, Minguillón et al. have investigated that *CDK5RAP3* may influences breast cancer in *BRCA1/2* mutation carriers by interacting with *BRCA2* and supporting DNA repair. CDK5RAP3 downregulation leads to reduced genomic instability, DNA damage resistance and increased tumour aggressiveness, potentially accelerating cancer progression in *BRCA1/2* mutation carriers. Additionally, it has been found that genetic variations in the *CDK5RAP3* locus are associated with breast cancer risk in *BRCA1/BRCA2* PV carriers, highlighting its role in cancer etiology [52].

Breast cancer in *BRCA2* pathogenic variants carriers is primarily ER-positive [53]. This means that SNPs associated with ER-positive breast cancer in general population, which represent the majority of susceptibility variants identified through GWAS, are at most associated with higher risk of developing disease in individuals carrying mutations in *BRCA2* gene [54]. Such an association with ER-positive tumours has been identified, among others, for: *FGFR2* (rs2981582), *TOX3/TNRC9* (rs3803662), *LSP1* (rs3817198) and *SLC4A7/NEK10* (rs4973768) (Table 3). There is also evidence that SNPs in RNA genes, such as *LINC02698* (rs2186703) and *LOC105373204* (rs55998524) are associated with lobular breast cancer for *BRCA2* mutation carriers [55].

For *BRCA2* PV carriers, only polymorphisms in *FGFR2* (rs2981582) and *TFAP2A* (rs9348512) reached GWAS significance threshold (*p*-value < 5.0 × 10^− 8^).

## Conclusions

This study presents a review of existing data on the impact of genetic modifiers on breast cancer risk among individuals carrying pathogenic variants in the *BRCA1* and *BRCA2* genes. GWAS have contributed significantly to the identification of breast cancer susceptibility variants in the general population. Importantly, research conducted by CIMBA recognizing some of these variants as modifiers of breast cancer risk in carriers of *BRCA1* and *BRCA2* pathogenic variants. The importance of these studies has been constantly increasing over the years and a greater number of research efforts focused on investigating the role of genetic modifiers [31].

An extensive knowledge about breast cancer risk modifiers, including genetic modifiers, has several benefits. One of the key advantages is improved risk stratification, which helps differentiate individuals with a high or low risk of developing breast cancer [56]. This allows for more personalized risk assessments rather than a ‘one-size-fits-all’ approach. With better risk prediction, screening and surveillance strategies can also be tailored more effectively. Lower-risk individuals may avoid unnecessary procedures, while those at higher risk can undergo more intensive screening. Additionally, personalized prevention strategies can be developed, including lifestyle modifications or chemoprevention, based on an individual’s specific risk profile. Also refined risk estimates may be helpful for carriers in making decisions, especially when it came to determining the timing of prophylactic surgeries [57, 58]. Some of the genetic modifiers identified have been already integrated into existing breast cancer risk prediction models. Among the SNPs discussed in our review, three variants (*TERT* rs10069690, *EMBP1* rs11249433, *FGF10/MRPS30* rs10941679) are incorporated in PRS_313_ [59]. PRS_313_ is a well-validated polygenic risk score for breast cancer in the general population, covering 313 breast cancer-associated variants. Its association with breast cancer risk has been demonstrated in multiple studies and resulted in its inclusion in cancer prediction models such as BOADICEA (Breast and Ovarian Analysis of Disease Incidence and Carrier Estimation Algorithm), Tyrer-Cuzick Model (IBIS Risk Evaluator) or Breast and Prostate Cancer Cohort Consortium (BPC3) Risk Model [59–61]. Additionally, PRS has been shown to result in absolute risk differences for the development of breast cancer in *BRCA1/2* PV carriers. In a study by Barnes et al. [62] PRS_313_ was significantly correlated with breast cancer risk (HR = 1.31, 95% CI [1.27–1.36]) among *BRCA2* PV carriers. Furthermore, the (ER)-negative PRS 313(which uses the same variants but with weights adapted to provide better prediction for ER-negative disease) was associated with breast cancer risk (HR = 1.29, 95% CI [1.25–1.33]) among *BRCA1* PV carriers. However, the effect was smaller than in the general population. Another study indicated that the estimated lifetime breast cancer risk for *BRCA1* and *BRCA2* PV carriers increased with higher PRS_313_ scores, though the observed effect was smaller than in the general population or among carriers of PVs in *ATM*, *CHEK2*, and *PALB2* [63]. Furthermore, PRS_313_ has demonstrated potential in refining contralateral breast cancer risk predictions for *BRCA1/2* PV carriers. For *BRCA1* heterozygotes, the (ER)-negative PRS_313_ showed the strongest association with contralateral breast cancer risk (HR = 1.12, 95% CI [1.06–1.18]), while for *BRCA2* heterozygotes, the ER-positive PRS_313_ was more strongly associated with contralateral breast cancer risk (HR = 1.15, 95% CI [1.07–1.25]) [64]. Despite these findings support the utility of PRS_313_ in risk prediction, it is essential to recognize that PRS-based screening programs require validation in prospective, randomized clinical trials. Ongoing studies, including Wisdom (ClinicalTrials.gov identifier: NCT02620852) and eMERGE in the United States, MyPeBS (ClinicalTrials.gov identifier: NCT03672331) in Europe, and PERSPECTIVE I&I in Canada, are currently exploring the effectiveness of PRS in breast cancer screening. The outcomes of these trials will ultimately determine whether PRS can enhance the personalization of breast cancer screening programs [65].

Another significant benefit is the potential for targeted therapies. Understanding how genetic modifiers influence breast cancer risk may result in identification of new molecular pathways that may serve as therapeutic targets. This could lead to more effective treatments for *BRCA1* and *BRCA2* PV carriers. Furthermore, studying genetic modifiers enhances the overall understanding of tumour biology, shedding light on the complex interactions that drive cancer development in these individuals [66]. Finally, identifying genetic modifiers can have a meaningful impact on psychosocial and reproductive decision-making. More precise risk information enables individuals to make informed choices about family planning and preventive measures, reducing uncertainty and anxiety [67, 68]. Overall, these benefits contribute to a more personalized and effective approach to healthcare for *BRCA1* and *BRCA2* carriers.

Although genetic risk modifiers appear promising for improving risk prediction, personalized prevention, and targeted therapies, several challenges must be addressed before they can be effectively integrated into clinical practice [66]. One major difficulty is related to complexity of genetic interactions. Breast cancer risk is influenced by multiple genetic and environmental factors, with genetic modifiers often having small individual effects. This makes it difficult to point their exact contributions. Additionally, interactions between different genes further complicate risk prediction, as the effect of one modifier may depend on the presence of another [69, 70]. Another challenge is the need for large sample sizes. Since genetic modifiers often have subtle effects, detecting them requires extensive and diverse study populations. Recruiting enough *BRCA1* and *BRCA2* mutation carriers for statistically significant findings is difficult, as they represent only a small subset of breast cancer patients. This limitation can slow down research progress and complicate the ability to draw definitive conclusion. Variability across populations also poses a problem. Genetic risk modifiers may differ among ethnic and ancestral groups, meaning that findings from one population may not be applicable to others [66, 71, 72]. This highlights the need for studies with broad, diverse representation or large studies within specific populations to ensure that risk models are inclusive and accurate for all individuals. Environmental and lifestyle factors further complicate research on genetic modifiers. Factors such as diet, exercise, and hormonal determinants can modify breast cancer risk, making it difficult to isolate the effect of specific genetic modifiers. These variables need to be taken into account to draw accurate conclusions [73]. Another major obstacle is the limited functional understanding of genetic modifiers. Even when they are identified through genome-wide association studies (GWAS), their biological role in cancer development is often unclear. Without a deeper understanding, it is challenging to translate genetic findings into actionable insights that can improve risk assessment and treatment strategies [74, 75].

Finally, despite growing evidence that polygenic risk scores PRS and other common genetic variants may modulate breast cancer risk, integrating this information into risk prediction models for *BRCA1/2* carriers is filled with challenges. The already high baseline risk in these individuals limits the relative impact of genetic modifiers, making it difficult to derive clinically meaningful stratification. Furthermore, the clinical utility of such refined risk estimates is not yet fully established. Current guidelines are primarily based on the presence of high-penetrance mutations, and the introduction of PRS-based stratification would require rigorous validation, standardization of scores, and clear demonstration of added predictive value. Additionally, ethical considerations, patient communication, and potential anxiety around more nuanced risk categories pose practical barriers.

## Data Availability

No datasets were generated or analysed during the current study.

## Abbreviations

PV: Pathogenic variant
SNP: Single nucleotide polymorphism
CNV: Copy number variation
GWAS: Genome–wide association study
WES: Whole exome sequencing
WGS: Whole genome sequencing
